# Paraneoplastic proteinuria in papillary renal cell carcinoma; a case report

**DOI:** 10.15171/jrip.2016.44

**Published:** 2016-08-03

**Authors:** Fatemeh Yaghoubi, Maliheh Yarmohammadi, Mohammad Vasei

**Affiliations:** ^1^Nephrology Research Center and Department of Nephrology, Tehran University of Medical Sciences, Tehran, Iran; ^2^Department of Nephrology, Semnan University of Medical Sciences, Semnan, Iran; ^3^Department of Pathology and Laboratory Medicine, Tehran University of Medical Sciences, Tehran, Iran

**Keywords:** Renal cell carcinoma, Paraneoplastic glomerulopathy, Nephrotic syndrome

## Abstract

We report a 55-year-old man presented with anemia and weakness, history of flank pain, hematuria and nephrotic syndrome. Spiral abdominopelvic computerized tomography (CT) scan showed multiloculated cystic mass (120 ×100 ×80 mm) in lower portion of left kidney with internal enhancing solid components and coarse peripheral calcifications. Radical nephrectomy of left kidney was done and biopsy confirmed renal cell carcinoma (RCC), papillary type, sarcomatoid foci, Fuhrman grade III. We assumed that, presence of nephrotic syndrome and paraneoplastic glomerulopathy leaded to heavy proteinuria in this case. Secondary, paraneoplastic glomerulopathy such as immunoglobulin A nephropathy and focal segmental glomerulosclerosis as a paraneoplastic syndrome of RCC have been reported previously. RCC can present with a wide range of signs and symptoms. Atypical presentations of papillary RCC such as proteinuria should be considered for patients presenting with nephrotic syndrome.

**Implication for health policy/practice/research/medical education:**

We report a 55-year-old man presented with anemia and weakness, history of flank pain, hematuria and nephrotic syndrome. Spiral abdominopelvic computerized tomography (CT) scan showed multiloculated cystic mass (120 ×100 ×80 mm) in lower portion of left kidney with internal enhancing solid components and coarse peripheral calcifications. This case report implicated that proteinuria should be considered as an atypical presentations of renal cell carcinoma (RCC).

## Introduction

Renal cell carcinoma (RCC) is a lethal tumor that accounts for 2%–3% of all adult malignancies and is the seventh most common cancer in men and the ninth most common cancer in women (1,2). In 2004, the World Health Organization (WHO) recognized 10 types of RCC based on morphologic and genetic characteristics, which clear cell adenocarcinoma is the most common (70%–85% adults) (1,3). Papillary RCC occurs in 7%-15% of adult patients with RCC (1).

Although diagnose of renal tumors (>50% cases) has significantly increased by widespread use of sophisticated imaging, a large number of patients with RCC still present with a wide array of symptoms such as flank pain, gross hematuria, palpable abdominal mass, metastatic symptoms and paraneoplastic syndromes (1,4). Some of the signs and symptoms of RCC are except from the rule of our awareness and understanding of this disease. Here we report a patient with papillary RCC diagnosis.

## Case Presentation

A 55-year-old man presented to the emergency department with flank pain, malaise and weakness since two months ago with exertional dyspnea and lower extremity edema. He had renal stone and flank pain from 12 years ago and a history of duodenal ulcer. He has been smoking one box per day for 20 years.

Urinalysis revealed hematuria with many red blood counts (RBC), white blood count (WBC) 10-12/HPPF and a daily proteinuria 3750 mg/day. Blood test showed hypochromic-microcytic anemia with hemoglobin 6.6 g/dl. Serum calcium and liver enzyme was in normal ranges. Hepatic viral marker and HIV antibody and wright were negative. Complement component (C3, C4, CH50), antinuclear antibody (ANA) and perinuclear antineutrophil cytoplasmic antibodies (p-ANCA) and cytoplasmic antineutrophil cytoplasmic antibodies (C-ANCA) were normal ranges. He went under upper and lower endoscopy for investigating of possible malignancy and the result was normal.

Spiral chest and abdominopelvic computerized tomography (CT) scan (without contrast) was conducted. Deformity and mass like appearance with lobulated border was seen mostly in lower pole of kidney associated with rim like calcified foci with probable cystic component which located in cortical and calyceal regions. Ureter seemed unremarkable. Perinephric fat was preserved. Significant adenopathy was noted (35 × 20 mm) in left renal hilum.

**Figure 1:**
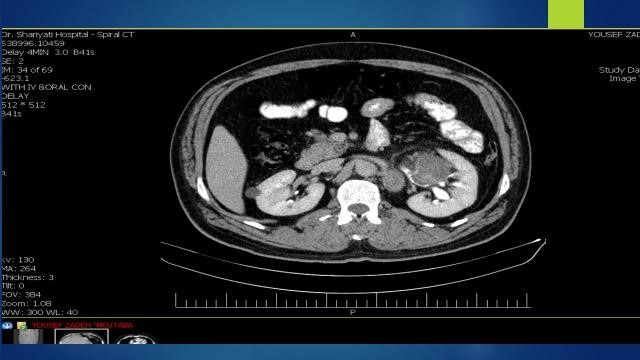
Computerized tomography scan of abdomen with contrast showing left renal mass.

Spiral abdominopelvic CT scan (with IV and oral contrast), showed; multi-loculated cystic mass (120 × 100 × 80 mm) in mid and lower portion of left kidney with mild internal enhancement and coarse peripheral calcifications in favor of RCC. A significant lymphadenopathy (32 × 20 mm) was visualized in left para aortic region. There were also two lymphadenopathies in left renal hilum (12 × 10 mm & 6 × 5 mm). Extension of mass to left perinephric space was seen. No gross extension to renal vein was found. Few bilateral small simple renal cortical cysts were visualized (Figure 1).

**Figure 2:**
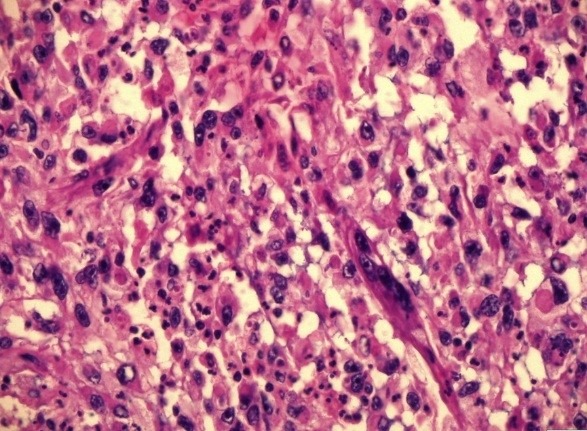
Sections of kidney showing papillary type carcinoma, sarcomatoid foci, Fuhrman grade III (×200).

Radical nephrectomy of left kidney was done and biopsy confirmed RCC, papillary type (12×9×6 cm), sarcomatoid foci (Fuhrman grade III; Figure 2).

## Discussion

The classic triad of palpable mass, hematuria, and flank pain occurs in less than 15% of patients with RCC while paraneoplastic syndrome may be the initial clinical presentation of RCC in a large number of patients (4,5). It is estimated that 10%–40% of patients with this disease will be involved a paraneoplastic syndrome and recognition of these syndromes may facilitate early diagnosis (5). Paraneoplastic syndromes associated with RCC can be classified to endocrine (hypercalcemia, hypertension, polycythemia, and Cushing syndrome) and nonendocrine (anemia, vasculopathy, coagulopathy and neuromyopathies) origins (6). Hypercalcemia is the most common of the paraneoplastic syndromes among patients with RCC and affect up to 20% of them (5).

As proteinuria did not associate with any other diseases and paraneoplastic glomerulopathy such as immunoglobulin A nephropathy and focal segmental glomerulosclerosis as a paraneoplastic syndrome of RCC have been reported previously (7,8) hence paraneoplastic glomerulopathy of RCC as a cause of heavy proteinuria could be a rational finding in this case.

Normal urinary protein excretion is <150 mg/24 hour (9). Urinary excretion >3.0 g/day is considered as heavy proteinuria and is usually a result of glomerular disease (10).

Causes of glomerular disease can be classified as primary (no evidence of extra-renal disease) or secondary (kidney involvement in a systemic disease) (11). Immunoglobulin A (IgA) nephropathy and primary focal segmental glomerulosclerosis are a primary proliferative glomerulonephritis and non-proliferative glomerulonephritis, respectively. Causes of proteinuria may be due to primary glomerular disease too. Also, secondary glomerular diseases may be associated with active urine sediment such as lupus nephritis, diabetic nephropathy, and amyloidosis and also hypertensive nephrosclerosis. Laboratory tests showed the lack of them in our case.

## Conclusion

RCC can present with a wide range of signs and symptoms. Atypical presentations of RCC should be considered for patients presenting with proteinuria of unknown origin.

## Authors’ contribution

Primary draft introduced by FY. MV contributed to the pathologic study and figures. MY participated in the case discussion. Manuscript edited by MY. All authors read the final version.

## Conflicts of interest

The authors declared no competing interests.

## Ethical considerations

Ethical issues (including plagiarism, data fabrication, double publication) have been completely observed by the authors. Written consent was obtained from the patient for publication of the study.

## Funding/Support

None.
